# Steady-state diode-laser photocrystallography at the P11 beamline (PETRA III): proof-of-concept measurements on an Fe(II) metallogrid complex

**DOI:** 10.1107/S1600577526009902

**Published:** 2026-10-30

**Authors:** Krishnayan Basuroy, Jose de Jesus Velazquez-Garcia, Sreeju Sreekantan Nair Lalithambika, Torben Reuss, Guillaume Pompidor, Alexander Grebentsov, Önder Akçaalan, Argha Barman, Simone Techert

**Affiliations:** a Photon Science Division Deutsches Elektronen-Synchrotron DESY Notkestraße 85 22607Hamburg Germany; b Institut für Anorganische Chemie Georg-August-Universität Göttingen Tammannstraße 4 37077Göttingen Germany; RIKEN SPring-8 Center, Japan

**Keywords:** photocrystallography, diode-laser excitation, synchrotron radiation, single-crystal X-ray diffraction, spin-crossover, Fe(II) metallogrids

## Abstract

Compact optical-excitation approaches have previously enabled synchrotron photocrystallography, including LED-pump/X-ray-multiprobe methods for sub-second time-resolved measurements. Here, we report the implementation of a compact pulsed diode-laser excitation platform for molecular single-crystal photocrystallography at the P11 beamline of PETRA III (DESY). The setup uses a 375 nm picosecond diode laser with compact focusing optics mounted on a fixed translation-stage assembly compatible with the P11 diffractometer and cryogenic sample environment. A [2 × 2] Fe(II) metallogrid complex was used as a model system to evaluate the experimental approach. Diffraction datasets were collected before laser irradiation and at selected delay times after termination of laser exposure. At 80 K, laser irradiation produced small but correlated changes in 〈Fe–N〉 distances, Fe⋯Fe separations, and photodifference maps, consistent with partial spin-state redistribution within the metallogrid framework. The response relaxed on the tens-of-minutes timescale and was not reproduced at 100 K, supporting assignment to a low-temperature metastable photoinduced structural state. Although some of the structural changes are modest and the photoconversion estimates should be regarded as qualitative indicators, the results demonstrate that compact pulsed diode-laser excitation can be integrated into the P11 single-crystal diffraction environment for steady-state molecular photocrystallography. The implementation also provides a practical foundation for future laser-synchronized pump–probe photocrystallography experiments at synchrotron beamlines.

## Abstract 2

A compact pulsed diode-laser excitation platform was implemented at the P11 beamline of PETRA III for steady-state molecular photocrystallography. Post laser irradiation single-crystal diffraction measurements on an Fe(II) metallogrid revealed small but correlated structural changes consistent with partial spin-state redistribution, demonstrating a practical route for diode-laser-based photocrystallography at synchrotron beamlines.

## 1. Introduction

Over the years, photocrystallography has become a well established method for directly observing structural changes in crystalline materials induced by light irradiation (Coppens, 2017 ▸; Cole, 2008 ▸; Hatcher *et al.*, 2024 ▸). Time-resolved pump–probe photocrystallography has emerged as a powerful approach for capturing the structural dynamics of molecules and crystalline motifs following photoexcitation, providing direct access to transient intermediates and reaction pathways in the solid state (Techert *et al.*, 2001 ▸; Kim *et al.*, 2002 ▸; Coppens, 2009 ▸; Hatcher & Raithby, 2014 ▸). Such studies have significantly advanced the understanding of ultrafast photoinduced processes in molecular crystals, including transition-metal complexes, purely organic chromophores, and molecular materials undergoing rapid electronic and structural reorganization on timescales ranging from nanoseconds to milliseconds (Coppens, 2015 ▸; Novozhilova *et al.*, 2003 ▸; Techert & Zachariasse, 2004 ▸; Basuroy *et al.*, 2017 ▸; Hatcher *et al.*, 2022 ▸; Basuroy *et al.*, 2023 ▸; Velazquez-Garcia *et al.*, 2024 ▸; Łaski *et al.*, 2024 ▸; Collet *et al.*, 2012 ▸).

Many photo-responsive molecular systems, however, undergo slower transformations that generate metastable states persisting for minutes to hours (Carducci *et al.*, 1997 ▸; Hatcher *et al.*, 2018 ▸; Marr *et al.*, 2023 ▸; Hatcher *et al.*, 2019 ▸). These long-lived photoinduced states are often more suitably investigated using steady-state or quasi-steady-state photocrystallographic approaches, where continuous illumination allows accumulation of the photoinduced species within the crystal lattice (Hatcher *et al.*, 2019 ▸). Structural data can then be collected under photo-stationary conditions, providing direct insight into the geometric and electronic changes associated with light-induced transformations. Such strategies are particularly valuable for systems exhibiting thermally reversible isomerization, spin-crossover transitions, and other cooperative phenomena in the crystalline state (Carducci *et al.*, 1997 ▸; Hayami *et al.*, 2000 ▸; Pillet *et al.*, 2012 ▸; Velazquez-Garcia *et al.*, 2022*a* ▸; Velazquez-Garcia *et al.*, 2022 ▸*b*; Velazquez-Garcia *et al.*, 2023 ▸).

While steady-state photocrystallography can often be implemented using relatively simple illumination schemes, time-resolved pump–probe experiments at synchrotron facilities generally require considerably more sophisticated optical infrastructure. Dedicated pump–probe beamlines frequently employ specialized laser systems, synchronization electronics, beam-conditioning optics, timing diagnostics, and optical beam-delivery paths to achieve reliable synchronization between optical excitation and X-ray probing. For example, laser facilities developed for time-resolved studies at BioCARS (APS) incorporate dedicated laser instrumentation, environmental stabilization, optical transport systems, and synchronization schemes specifically designed for pump–probe X-ray experiments (Graber *et al.*, 2011 ▸; Henning *et al.*, 2024 ▸). Although such facilities provide state-of-the-art capabilities for time-resolved measurements, their implementation and operation require substantial experimental infrastructure. Consequently, the development of compact, portable, and easily deployable photoexcitation platforms remains of considerable interest, both for steady-state photocrystallography and as a foundation for future time-resolved studies at synchrotron beamlines. Such systems may facilitate implementation of synchronized pump–probe methodologies at beamlines where dedicated laser infrastructure is not permanently available and can provide a practical route towards broader adoption of time-resolved photocrystallography.

Recently, Hatcher, Raithby and co-workers demonstrated access to the sub-second regime using an LED-pump/X-ray-multiprobe approach to investigate photoactivated NO_2_ → ONO linkage isomerization in [Pd(Bu_4_dien)(NO_2_)][BPh_4_] (Hatcher *et al.*, 2022 ▸). Their study highlighted the potential of compact and experimentally accessible photoexcitation sources as alternatives to conventional high-power laser systems for selected photocrystallographic applications.

In the present work, we implemented a compact diode-laser-based photocrystallography platform at the P11 beamline of PETRA III (DESY). Optical excitation was provided by a 375 nm DeltaDiode DD-375L pulsed laser diode (HORIBA Scientific), delivering approximately 50 ps pulses at repetition rates up to 80 MHz under standard operation. The compact and modular design of the system enables straightforward integration with synchrotron diffractometers and cryogenic sample environments while enabling efficient photoexcitation of the crystal before diffraction measurements. As a proof-of-concept application, we investigated a tetranuclear Fe(II) metallogrid complex exhibiting spin-crossover behaviour and explored the structural consequences of photoinduced spin-state redistribution at low temperature. The study demonstrates the suitability of compact diode-laser platforms for synchrotron-based steady-state photocrystallography and provides a practical framework for future implementation of pump–probe methodologies at beamlines where extensive laser infrastructure may not be readily available.

## 2. Experimental methods

### 2.1. Sample preparation and single crystal X-ray diffraction

The [2 × 2] Fe(II) metallogrid complex was synthesized and diffraction quality single crystals were grown following established procedures (Velazquez-Garcia *et al.*, 2022*a* ▸; Velazquez-Garcia *et al.*, 2022*b* ▸; Schneider *et al.*, 2013 ▸). Single crystals suitable for single-crystal X-ray diffraction (SCXRD) and photocrystallography experiments were selected and mounted on the goniometer. All the SCXRD experiments were performed at 80 K and 100 K. The X-ray data sets were collected in φ scan mode, using a monochromatic X-ray beam with λ = 0.82655 Å (15 keV) at the P11 beamline at PETRA III, DESY, Hamburg, Germany. All regular SCXRD and photocrystallography datasets were processed using the *XDS* program package (Kabsch, 1993 ▸). The structures from the regular SCXRD data were solved using *WinGX* (Farrugia, 2012 ▸), with the *SHELXT* (Sheldrick, 2015*a* ▸) structure solution program, and refined against *F*^2^ isotropically, followed by full matrix anisotropic least-squares refinement by *SHELXL* (Sheldrick, 2015*b* ▸). In the final cycle of refinement, hydrogen atoms were fixed geometrically in idealized positions and allowed to ride with the respective C atoms to which each was bonded.

### 2.2. Photocrystallography

**Figure 1:**
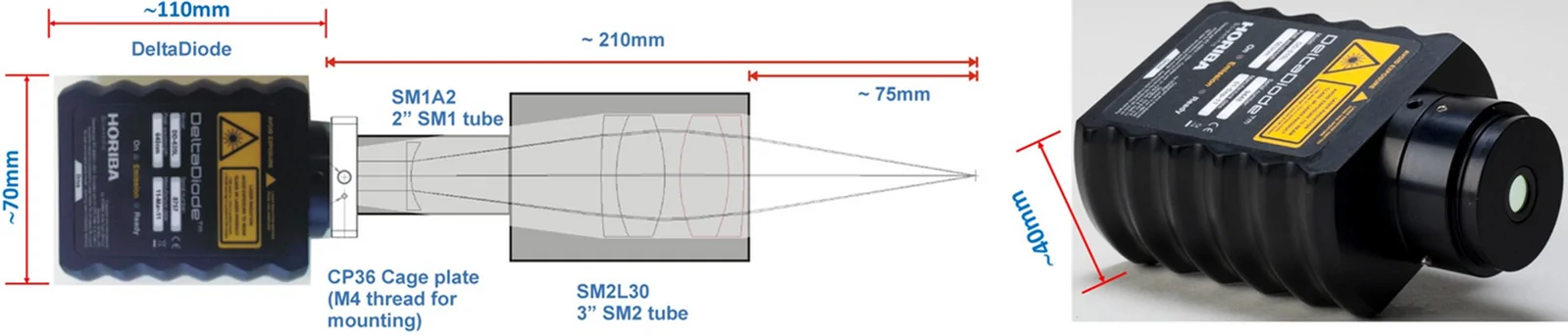
Dimensions of the diode laser and the associated focusing optics (figure supplied by HORIBA Scientific) attached to it.

**Figure 2:**
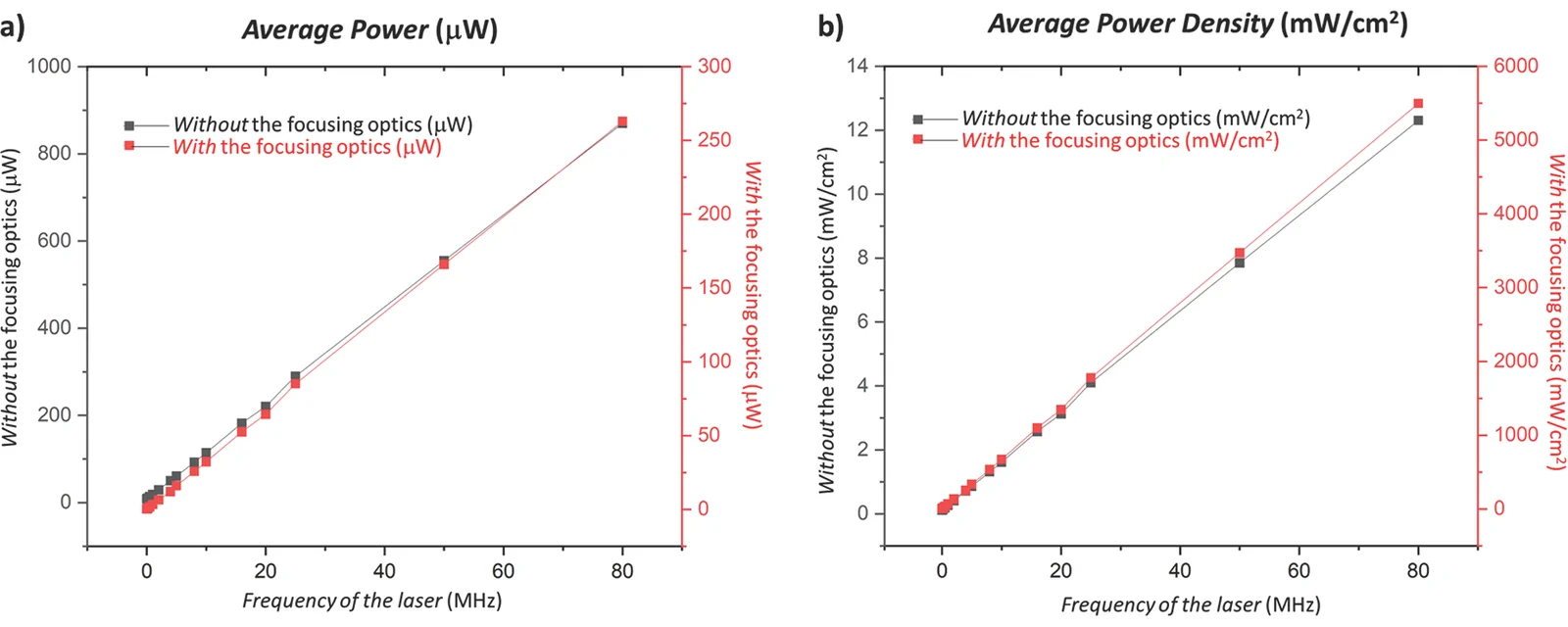
Characterization of the 375 nm diode-laser excitation system used for photocrystallography experiments at P11. (*a*) Average laser power measured as a function of repetition rate before and after the focusing optics. (*b*) Corresponding average power density at the sample position.

The photocrystallography experiments were conducted at the P11 beamline, DESY. The P11 beamline is equipped with two Kirkpatrick–Baez (KB) mirror systems (Kirkpatrick & Baez, 1948 ▸); the first system consists of two horizontally and one vertically deflecting KB mirrors (Burkhardt *et al.*, 2016 ▸). The refocusing system close to the sample position has also one horizontally and one vertically focusing KB mirrors. These dynamically bendable KB mirrors are quite capable of generating beam size of 4 µm × 9 µm at the sample position at 73.30 m from the source. By narrowing the secondary source aperture, beam sizes down to 1 µm × 1 µm with photon fluxes exceeding 2 × 10^1^^1^ photons s^−1^ can be routinely achieved in less than one minute, though at the expense of overall flux. For steady-state photocrystallography, exact pump–probe spatial overlap is less restrictive than in time-resolved pump–probe experiments; however, the relative sizes of the optical pump beam, X-ray probe beam and crystal remain important for efficient capture of the photoexcited state species. A class 3B diode laser equipped with compact compound-lens focusing optics was employed (Fig. 1 ▸ and Table S1 of the supporting information). The compound-lens focusing system was supplied by HORIBA Scientific. The average laser power and average laser power density, with and without the focusing optics, were measured using a power meter and the results are shown in Fig. 2 ▸ (Table S2). The output power of the 375 nm DeltaDiode DD-375L laser was characterized over the full range of accessible operating repetition rates. During power characterization, only minor fluctuations in the measured average power were observed at fixed repetition rates, indicating stable operation over the duration of the measurements. Average laser power was measured before and after the focusing optics, and the corresponding power densities at the sample position were calculated from the measured beam dimensions. As expected, the transmitted power decreased after the focusing optics because of optical losses associated with the beam-conditioning and focusing elements. However, focusing of the laser beam resulted in a more than 400-fold increase in average power density at the sample/crystal position, reaching values of approximately 5.5 × 10^6^ µW cm^−2^ at the maximum repetition rate of 80 MHz. The measurements quantify the dependence of laser power and power density on repetition rate under the experimental conditions employed at P11.

**Figure 3:**
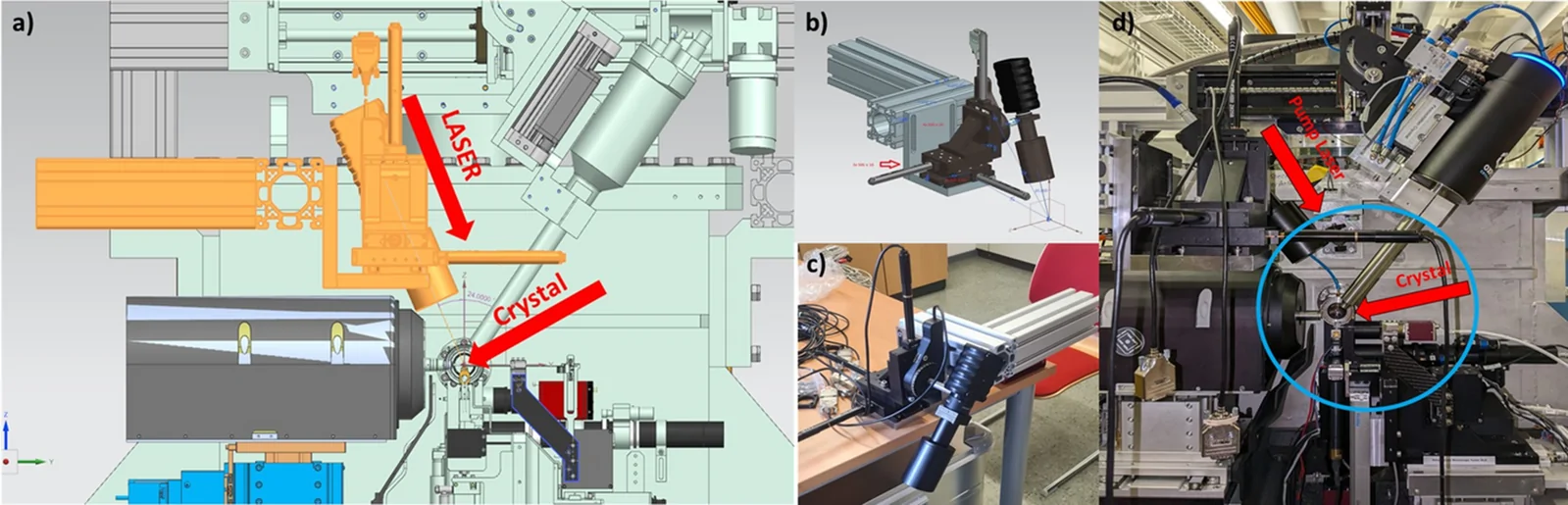
(*a*) Schematic representation of the photocrystallography setup with the class 3B diode laser adapted to the P11 beamline at DESY. (*b*) Schematic of the diode-laser/focusing-optics assembly mounted on a motorized Thorlabs multi-axis positioning system. The motorized axes were controlled using KDC101 K-Cube DC-servo controllers housed in a KCH601 controller hub. (*c*) Photograph of the laser setup attached to the motorized positioning assembly. (*d*) Photograph of the final setup after mounting on the P11 beamline.

For the present measurements, the diode laser was operated using its internal trigger at a repetition rate of 80 MHz. A motorized Thorlabs multi-axis positioning assembly was incorporated into a custom mounting frame for integration with the P11 beamline. The positioning system was driven by KDC101 K-Cube DC-servo motor controllers mounted in a KCH601 six-channel controller hub, enabling controlled adjustment of the diode-laser/focusing-optics assembly relative to the diffractometer and sample environment. Fig. 3 ▸ shows the schematic representation and photographs of the diode-laser/focusing-optics assembly mounted on the motorized Thorlabs multi-axis positioning system. Although the custom mounting frame was designed to accommodate the geometrical constraints of the P11 experimental hutch, the overall modular assembly can be adapted for implementation at other synchrotron beamlines.

The laser power densities listed in Table S2 and shown in Fig. 2 ▸ correspond to average irradiances calculated from the measured average laser power and illuminated beam area. These values should therefore be interpreted as indicators of steady optical loading at the sample surface and not as peak irradiances associated with individual laser pulses.

In recent years, a limited number of time-resolved studies have been conducted at the P11 beamline. Time-resolved pump–probe X-ray absorption fine structure spectroscopy experiments have been reported previously at P11 (Göries *et al.*, 2016 ▸; Naumova *et al.*, 2018 ▸). In addition, a time-resolved serial synchrotron crystallography study performed in 2019 employed 18 time points spanning from 30 ms to 30 s to visualize site-specific reactivity and correlated molecular breathing motions in the enzyme fluoro­acetate dehalogenase (Mehrabi *et al.*, 2019 ▸). Despite these developments, optical laser pump–hard X-ray probe photocrystallography experiments on molecular single crystals have not yet been implemented at the P11 beamline. In addition to performing steady-state photocrystallography measurements on molecular crystals containing long-lived photoinduced species with lifetimes ranging from minutes to hours, the present work also explores the feasibility of integrating an optical pump laser system at P11 that may support future pump–probe time-resolved photocrystallography experiments aimed at investigating transient photoinduced states.

Although the experiments reported here were performed under steady-state conditions, the laser platform was designed with future pump–probe applications in mind. The P11 beamline can operate in the PETRA III 40-bunch timing mode, where consecutive X-ray pulses are separated by 192 ns and have durations of approximately 100 ps (FWHM) (PETRA III, 2024 ▸). The DeltaDiode DD-375L laser supports external triggering up to 100 MHz and can therefore be synchronized with the storage-ring timing structure. Future implementation may also benefit from detector systems such as the LAMBDA detector from X-Spectrum, a hybrid photon-counting detector, which offers high frame rates and digital gating capabilities relevant to time-resolved synchrotron studies (Pennicard *et al.*, 2014 ▸; Ballabriga *et al.*, 2013 ▸). Under such conditions, the achievable temporal resolution is determined primarily by the convolution of the laser and X-ray pulse durations. Assuming pulse widths of Δ*t*_pump_ ≃ 50 ps for the laser and Δ*t*_probe_ ≃ 103 ps for the X-ray probe, an overall time resolution for Gaussian pulse profiles can be expected to be Δ*t* = √(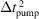 + 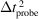) ≃ 115 ps. These considerations indicate that the present implementation provides a practical foundation for future time-resolved pump–probe photocrystallography experiments at P11 aimed at investigating transient photoinduced structural states.

**Figure 4:**
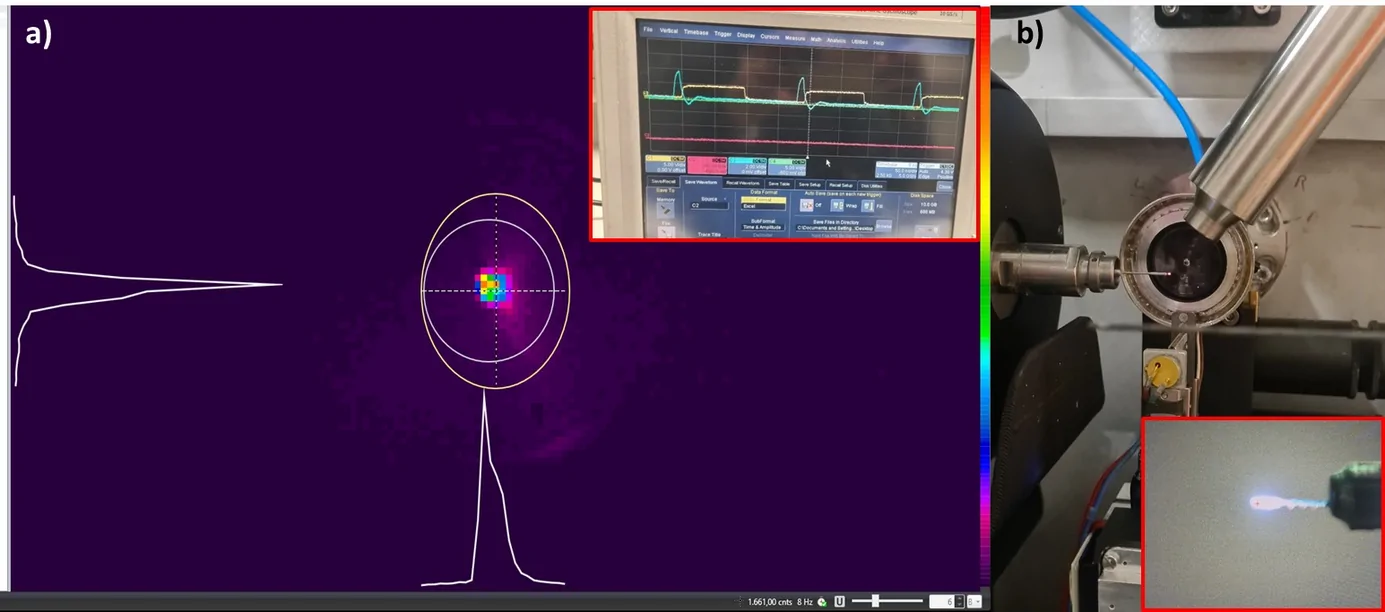
(*a*) Beam cross-section of the diode laser measured using an Ophir SP620U beam-profiling camera. Inset: oscilloscope traces of the laser-controller and PETRA III master-clock timing-reference signals, illustrating the synchronization concept for future pump–probe implementation. (*b*) Experimental arrangement showing the mounted single crystal under cryogenic conditions during laser irradiation at P11. Inset: photograph of the Fe(II) metallogrid crystal during laser irradiation, before the photocrystallography measurements.

With the compact focusing optics installed, the pulsed diode laser beam could be focused down to approximately 70 µm × 87 µm [Fig. 4 ▸(*a*) and Fig. S1]. Although a fast photodiode and pulse generator, which would enable direct visualization of the externally triggered laser output at the synchrotron pulse frequency and synchronization of the laser with the X-ray pulse train, were not available during the experiment, an indirect assessment of the pump–probe timing scheme was successfully achieved. Signals from the laser controller and the PETRA III master clock were simultaneously displayed on an oscilloscope, allowing visualization of the corresponding timing-reference signals. This arrangement provided an initial assessment of the relative timing scheme and synchronization feasibility for future pump–probe measurements, but did not constitute a direct fast-photodiode measurement of the optical laser pulse or direct measurement of the X-ray pulse profile. Fig. 4 ▸(*b*) shows the single crystal mounted on the goniometer in the beamline during irradiation with the focused diode laser beam.

**Figure 5:**
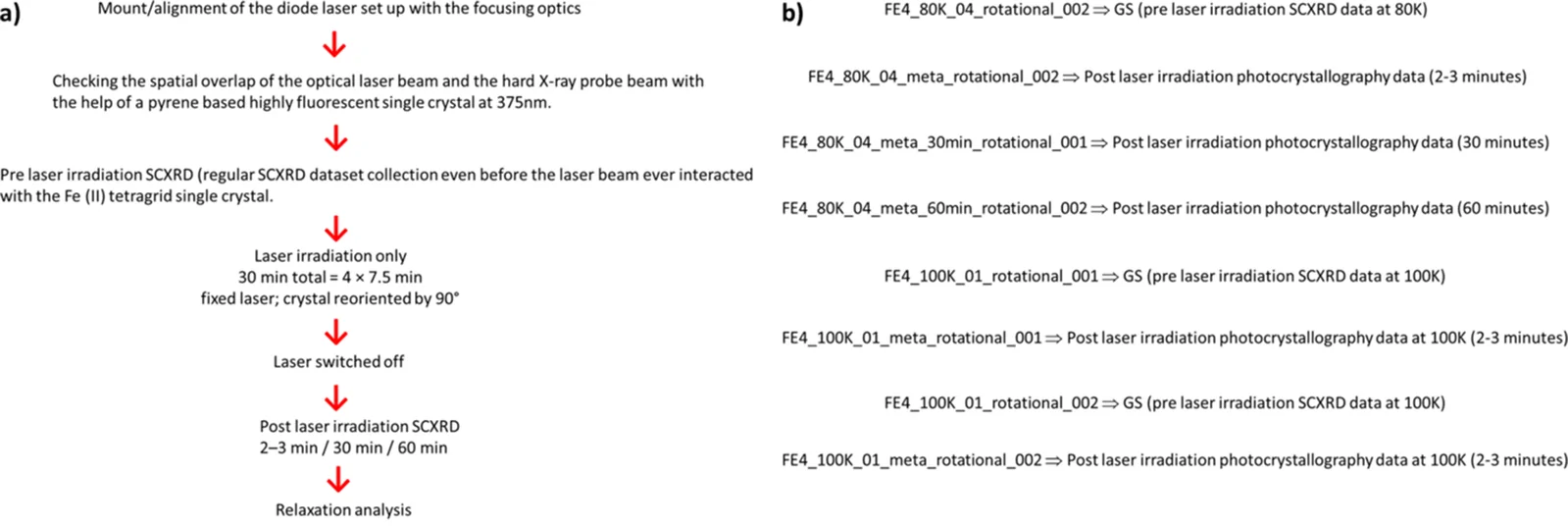
(*a*) Schematic representation of the experimental sequence used for the pre laser irradiation and post laser irradiation single-crystal X-ray diffraction measurements. A pre laser irradiation diffraction dataset was first collected with the laser off. The crystal was then irradiated for 30 min in total using the fixed 375 nm diode-laser setup. To improve illumination uniformity, the crystal was reoriented relative to the fixed laser beam in four orientations separated by 90°, with 7.5 min irradiation at each orientation. After the laser irradiation was terminated, diffraction datasets were collected at selected delay times to monitor the relaxation of the photoinduced structural state. Panel (*b*) identifies each dataset filename together with its corresponding temperature, irradiation condition and post laser irradiation delay time.

After mounting the diode laser equipped with the focusing optics on the beamline, a highly fluorescent pyrene-based molecular crystal was used to spatially align the laser beam at the sample position. The average crystal dimensions used for alignment were approximately 50–100 µm. Subsequently, single crystals of the Fe(II) metallogrid complex were mounted and aligned with respect to both the X-ray beam and the diode laser. Once the precise focusing of the laser beam at the sample position was achieved, the laser setup remained in a fixed orientation for the entirety of the measurements. The crystals were irradiated for a total of 30 min in four 7.5 min periods. Between successive irradiation periods, the crystal mounted on the goniometer was rotated by 90° relative to the fixed laser beam, thereby providing irradiation from four different directions to improve illumination uniformity. The diode laser was operated at its internally triggered repetition rate of 80 MHz during the measurements. A series of diffraction datasets were collected following termination of laser irradiation. The acquisition time for a 180° φ-scan was approximately 39 s, while additional experimental procedures, including access to the hutch and sample handling, introduced an additional delay of approximately 2–3 min after the irradiation had ceased. Data were collected at 80 K and 100 K, and datasets were acquired both before laser irradiation and at regular time intervals after the termination of laser exposure to monitor the structural evolution and relaxation behaviour of the photoinduced state within the same crystal. Fig. 5 ▸ describes how the pre laser irradiation SCXRD datasets and post laser irradiation/photocrystallography datasets were collected.

All diffraction datasets were processed using the *XDS* package, including indexing, integration, scaling and merging of symmetry-related reflections. Structure solution and refinement were carried out in *WinGX*, employing *SHELXT* for structure solution and *SHELXL* for refinement against *F*^2^. Initial isotropic refinement was followed by anisotropic refinement of all the non-hydrogen atoms. Owing to the limited data quality after irradiation, reduced completeness associated with rapid data collection, partial photoconversion, and the presence of positional disorder, disorder modelling using partial occupancies could not be achieved for some of the non-hydrogen atoms that would otherwise have been warranted. The photodifference maps, which show differences in electron density following laser irradiation, were prepared from pairs of *SHELXL* LIST6 FCF files. This format was used because it provides both measured 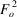 values and calculated phases for each reflection. Common reflections between the ground state/pre laser irradiation and post laser irradiation datasets were matched, observed amplitudes were obtained as *F* = √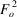, and a global scale factor *k* was applied to the ground-state or pre laser irradiated amplitudes. Difference coefficients were calculated as Δ*F* = *F*_post_ − *kF*_pre_. Ground-state or pre laser irradiated calculated phases were used for map generation; for negative Δ*F* values, the phase was shifted by 180° and |Δ*F*| was used as the coefficient. The generated pseudo-FCF files were used only for qualitative visualization of photodifference density. Photodifference maps were visualized in *Olex2* using the ground-state structural model and the generated pseudo-FCF file (Dolomanov *et al.*, 2009 ▸). Where necessary, symmetry-equivalent atoms were generated to display the complete metallogrid molecule. BF_4_^−^ counterions were hidden only for graphical clarity and were not removed from the refinement model.

## 3. Results and discussion

Photocrystallography measurements were performed using several delay intervals after termination of the laser irradiation. In the present study, single-crystal X-ray diffraction datasets were collected at approximately 2–3 min, 30 min, and 60 min after stopping the laser exposure. Measurements before and after irradiation were performed using the same crystals. Although the overall structural trends remained consistent, some variations were observed, particularly in parameters such as 〈Fe–N〉 bond lengths, Fe⋯Fe separations at the LS and HS sites, and the distortion parameters Σ and Θ. The definitions and structural significance of these parameters have been discussed previously (Velazquez-Garcia *et al.*, 2024 ▸; Velazquez-Garcia *et al.*, 2022*a* ▸; Velazquez-Garcia *et al.*, 2022*b* ▸; Velazquez-Garcia *et al.*, 2023 ▸). At each time of data collection following the termination of the laser irradiation, the diffraction data represent an ensemble average containing contributions from both the ground-state 2HS–2LS configuration and laser-induced species. The observed increase in 〈Fe–N〉 bond lengths was therefore used as an indicator to estimate the relative population of the laser-induced state, which is suggestive of partial redistribution toward a 3HS–1LS configuration.

**Figure 6:**
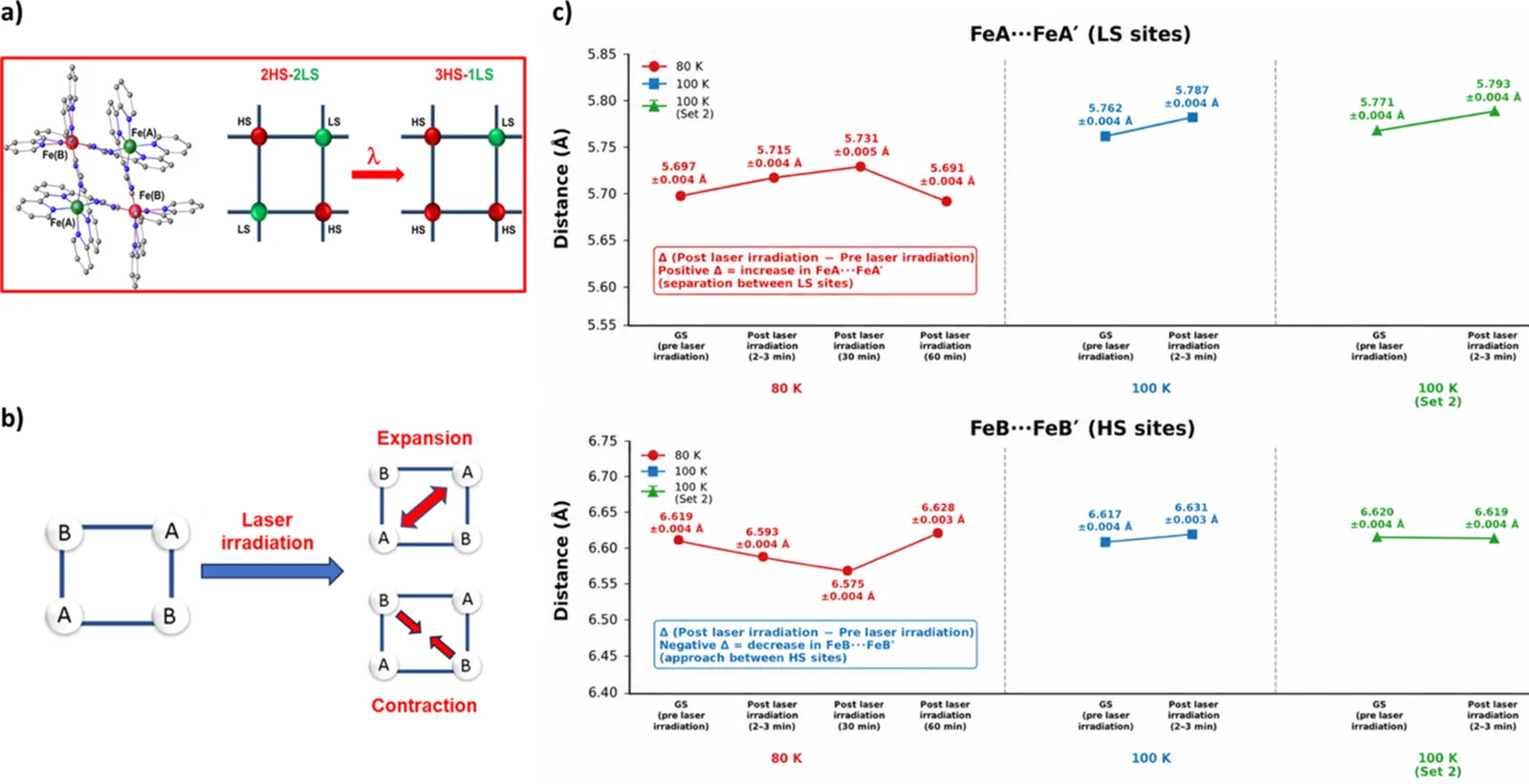
(*a*) Schematic representation of the structural consequences expected for a photoinduced redistribution from the 2HS–2LS ground state towards a 3HS–1LS configuration previously reported for this Fe(II) tetragrid system. (*b*) The conversion of one LS site to an HS site is accompanied by expansion of the Fe*A*⋯Fe*A*′ separation and contraction of the Fe*B*⋯Fe*B*′ separation. (*c*) These panels show the experimentally observed evolution of these distances following laser irradiation.

Upon irradiation, photoinduced structural signatures suggestive of partial redistribution from the initial 2HS–2LS state toward a transient 3HS–1LS population were observed [Fig. 6 ▸(*a*)]. These changes are reflected in variations of the 〈Fe–N〉 bond lengths, modifications of the Fe⋯Fe separations [Fig. 6 ▸(*c*)], and distortions within the Fe(II) metallogrid framework. The Fe⋯Fe distances together with the coordination geometry parameters were analysed using the *OctaDist* program to monitor possible spin-state evolution (Ketkaew *et al.*, 2021 ▸). Selected structural parameters are summarized in Table S3, while the complete *OctaDist* outputs generated from the CIF files are provided as Table S4. For the present Fe(II) metallogrid system, the experimentally observed difference between the 〈Fe–N〉 bond lengths at the LS and HS sites was approximately 0.1 Å, whereas a larger separation of approximately 0.2 Å has been reported previously for related measurements (Velazquez-Garcia *et al.*, 2024 ▸). The smaller LS–HS bond-length separation observed here may reflect differences in data quality/completeness, differences in refinement strategy, or crystal-to-crystal variation. Previous work by Velazquez-Garcia *et al.* reported temperature-dependent variations in the average Fe–N distances of this metallogrid family over the 80–100 K range during gradual warming (Velazquez-Garcia *et al.*, 2022*c* ▸). Since the present measurements indicate an approximate 0.1 Å separation between the LS and HS states, the following expression was used as a qualitative measure to estimate the relative photoconversion,
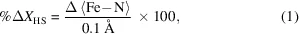
where Δ〈Fe–N〉 denotes the variation in the average metal–ligand bond length associated with the laser-induced response. The value of 0.1 Å corresponds to the experimentally observed LS–HS bond-length difference for the present system. As the laser-induced changes in 〈Fe–N〉 remain comparable with their associated experimental uncertainties, the calculated values should be regarded only as indicative of possible bond expansion and partial photoconversion rather than absolute conversion percentages. Fig. 6 ▸(*c*) shows the experimentally observed changes in the non-bonded Fe⋯Fe separations at the LS and HS sites. During the photoinduced response suggestive of 2HS–2LS → 3HS–1LS redistribution, the Fe*A*⋯Fe*A*′ distances between the LS sites generally increase, whereas the corresponding separations between the HS sites tend to decrease [Fig. 6 ▸(*c*) and Table S3].

**Figure 7:**
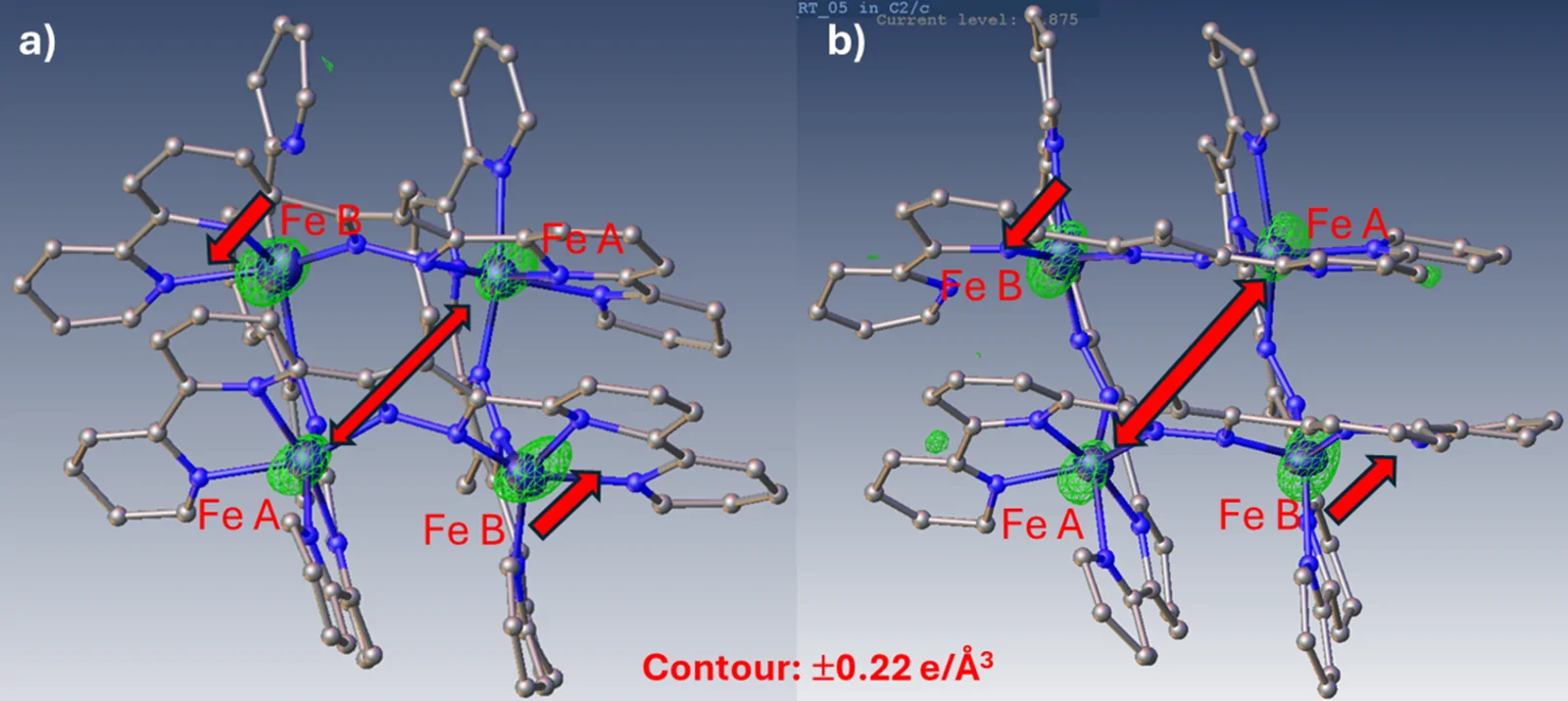
Photodifference maps calculated between the pre laser irradiated (single-crystal X-ray diffraction datasets collected *before* laser irradiation) and post laser irradiated datasets (single-crystal X-ray diffraction datasets collected *after* laser irradiation) for (*a*) FE4_80K_04_Meta_rotational_002 (common reflections: 1519; scale factor *k*: 0.895785) and (*b*) FE4_80K_04_Meta_30min_rotational_001 (common reflections: 1254; scale factor *k*: 0.963260). The maps show displacement features consistent with increased separation of the Fe*A* sites supporting the distortion pattern proposed in Fig. 6 ▸. Displacement features at the Fe*B* sites indicate a lateral twisting motion approximately perpendicular to the plane shown.

This opposing displacement leads to distortion of the tetragrid framework. Although the angular distortion parameters do not show a direct one-to-one correlation with the proposed spin-state redistribution, the collective structural changes observed in the 〈Fe–N〉 distances, Fe⋯Fe separations, and photodifference maps consistently support the structural evolution. The photodifference maps obtained from the ground-state and laser-induced metastable-state datasets further indicate a lateral twisting motion at the HS sites, while the LS sites exhibit increased separation, consistent with the structural trends summarized in Table S3 (Fig. 7 ▸).

The photoconversion values estimated using the previously described expression are summarized in Table S5, yielding estimated relative photoconversion indicator values of approximately 23% and 9% for the 2–3 min and 30 min datasets, respectively, after irradiation. Although the absolute bond-length variations remain comparable with the associated e.s.d. values, the correlated evolution of 〈Fe–N〉, Fe*A*⋯Fe*A*′, and the time-dependent relaxation behaviour collectively support partial laser-induced redistribution from the initial 2HS–2LS state towards a metastable 3HS–1LS population (Table S3). For the dataset collected after 60 min, the increase in 〈Fe–N〉 at the Fe*A* (LS) site was no longer observed, while the expanded Fe*A*⋯Fe*A*′ separation relaxed towards the pre laser irradiation values [Fig. 6 ▸(*c*) and Table S3]. These observations are consistent with relaxation of the proposed metastable spin-state population towards the initial 2HS–2LS configuration. The persistence of these structural signatures over tens of minutes supports assignment to a metastable photoinduced spin-state population rather than to a short-lived electronically excited state. More importantly, the two Fe⋯Fe vectors show oppositely directed changes at 80 K: Fe*A*⋯Fe*A*′ expands while Fe*B*⋯Fe*B*′ contracts. The propagated-error analysis of the Fe⋯Fe separations was performed using σΔ = √(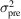 + 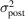), and the results are summarized in Table S6. At 80 K, the post laser irradiation datasets collected after 2–3 min and 30 min show statistically significant and oppositely directed changes in the Fe⋯Fe separations: Fe*A*⋯Fe*A*′ expands, whereas Fe*B*⋯Fe*B*′ contracts. The propagated-error analysis also confirms that both changes exceed their propagated standard uncertainties in the 2–3 min and 30 min datasets. This coupled distortion provides stronger structural evidence for a photoinduced rearrangement of the metallogrid than the small Fe–N bond-length changes considered alone [Fig. 6 ▸(*c*); Tables S3 and S6]. The corresponding |Δ|/σΔ values are approximately 3.2 and 4.6 for the 2–3 min dataset, and 5.3 and 7.8 for the 30 min dataset, respectively. In contrast, the 60 min dataset no longer shows significant Fe⋯Fe changes, consistent with relaxation towards the pre laser irradiation structure.

Additional post laser irradiation/steady-state photocrystallography measurements were also performed at 100 K; however, at 100 K, although small Fe*A*⋯Fe*A*′ increases are observed, the characteristic coupled Fe*A*⋯Fe*A*′ expansion/Fe*B*⋯Fe*B*′ contraction seen at 80 K is not reproduced [Fig. 6 ▸(*c*) and Table S3] and neither the 〈Fe–N〉 distances exhibited correlated changes following laser irradiation. This supports the assignment of the 80 K response to a metastable photoinduced distortion rather than to a uniform lattice expansion or a non-specific refinement artefact. The observation also suggests a temperature-dependent relaxation process rather than crystal-to-crystal variability. The higher temperature is therefore likely to promote faster thermal relaxation from the photoinduced state back to the equilibrium 2HS–2LS configuration, preventing measurable accumulation of the metastable species.

To further assess whether the observed changes could arise from non-specific heating or radiation-damage effects, equivalent isotropic *B* factors were calculated from the *U*_eq_ values reported in the CIF files using *B*_eq_ = 8π^2^*U*_eq_ (Table S7). The post laser irradiation datasets show moderate increases in average *B*_eq_ values, particularly for the disordered BF_4_^−^ counterions. However, comparable increases in *B*_eq_ are also observed in the 100 K post laser irradiation datasets, where no analogous correlated Fe⋯Fe structural response is observed. The *B*-factor analysis therefore does not by itself prove the origin of the structural changes, but supports the conclusion that the 80 K response is not simply a consequence of a uniform increase in atomic displacement parameters.

To further evaluate whether the observed structural response could be attributed to uniform lattice expansion or progressive deterioration of the diffraction data, unit-cell volumes and selected data-quality and refinement indicators were compared across the pre- and post laser irradiation datasets (Table S8). At 80 K, the unit-cell-volume changes were non-monotonic, corresponding to +0.258%, −0.018% and +0.367% at 2–3 min, 30 min and 60 min, respectively, relative to the pre laser irradiation dataset. Notably, the strongest coupled Fe*A*⋯Fe*A*′ expansion and Fe*B*⋯Fe*B*′ contraction occurred in the 30 min dataset, for which the unit-cell volume was nearly unchanged in relative terms, whereas the 60 min dataset showed the largest volume increase after the Fe⋯Fe response had substantially relaxed. Similar volume increases were also observed in the 100 K post laser irradiation datasets, where the characteristic coupled Fe⋯Fe response was not reproduced. The diffraction and refinement indicators did not show a uniform deterioration across the dataset series: although *R*_int_ increased with post laser irradiation delay at 80 K, *R*_1_ and *wR*_2_ remained below their respective pre laser irradiation values. Although these indicators do not by themselves exclude heating or radiation damage, they do not support a simple relationship between uniform lattice expansion or progressive diffraction deterioration and the specific coupled Fe⋯Fe distortion observed at 80 K.

The present work demonstrates the implementation of a compact pulsed diode-laser excitation platform at the P11 beamline of PETRA III (DESY) for steady-state molecular photocrystallography/post laser irradiation molecular photocrystallography. A tetranuclear Fe(II) metallogrid complex was used as a model system to evaluate the experimental approach. At 80 K, laser irradiation produced small but correlated changes in 〈Fe–N〉 bond lengths, Fe⋯Fe separations and photodifference maps. These structural signatures persisted for several tens of minutes before relaxing towards the pre laser irradiation state, whereas at 100 K, the characteristic coupled Fe*A*⋯Fe*A*′ expansion and Fe*B*⋯Fe*B*′ contraction observed at 80 K was not reproduced. The temperature dependence and relaxation behaviour are consistent with the formation of a metastable laser-induced structural state at low temperature. Although the bond-length variations are modest and the estimated photoconversion values should be regarded as qualitative indicators, the correlated evolution of multiple structural parameters supports the presence of a laser-induced response.

The present implementation is complementary to previously reported compact optical-pump approaches, particularly the LED-pump/X-ray-multiprobe method of Hatcher, Raithby and co-workers. In that approach, broad pulsed LED illumination combined with gated detector readout enables millisecond-to-second pump–multiprobe crystallography with uniform sample excitation. In contrast, the P11 implementation reported here uses a focused 375 nm picosecond diode-laser source operated at high repetition rate. Moreover, the focused diode-laser beam provides a compact and directional excitation geometry compatible with the spatial constraints of the P11 diffractometer and cryogenic sample environment. Although the present optical assembly was designed and characterized for excitation near 375 nm, the modular arrangement can accommodate other DeltaDiode laser heads, subject to the wavelength compatibility of the custom focusing optics. The high-repetition-rate and external-triggering capabilities of the laser provide a practical route towards future synchronization with the PETRA III timing structure.

Beyond the specific Fe(II) metallogrid investigated here, the results show that compact diode-laser excitation can be integrated into synchrotron single-crystal diffraction experiments without the need for a dedicated large-scale laser endstation. The small footprint, cryogenic compatibility and modular implementation of the setup make it suitable for steady-state photocrystallographic studies of photo-responsive molecular crystals at P11 and, with suitable adaptation, at other synchrotron beamlines. Its external-triggering capability also provides a basis for future synchronized time-resolved pump–probe photocrystallography at PETRA III and other facilities with compatible timing structures.

## Supplementary Material

Crystal structure: contains datablock(s) I. DOI: 10.1107/S1600577526009902/yi5197sup1.cif

Crystal structure: contains datablock(s) FE4_80K_04_meta_rotational_002. DOI: 10.1107/S1600577526009902/yi5197sup2.cif

Crystal structure: contains datablock(s) FE4_80K_04_meta_30min_rotational_001. DOI: 10.1107/S1600577526009902/yi5197sup3.cif

Crystal structure: contains datablock(s) FE4_80K_04_meta_60min_rotational_002. DOI: 10.1107/S1600577526009902/yi5197sup4.cif

Crystal structure: contains datablock(s) FE4_100K_01_rotational_001. DOI: 10.1107/S1600577526009902/yi5197sup5.cif

Crystal structure: contains datablock(s) FE4_100K_01_rotational_002. DOI: 10.1107/S1600577526009902/yi5197sup6.cif

Crystal structure: contains datablock(s) FE4_100K_01_meta_rotational_001. DOI: 10.1107/S1600577526009902/yi5197sup7.cif

Crystal structure: contains datablock(s) FE4_100K_01_meta_rotational_002. DOI: 10.1107/S1600577526009902/yi5197sup8.cif

Structure factors: contains datablock(s) I. DOI: 10.1107/S1600577526009902/yi5197sup9.hkl

Figure S1 and Tables S1 to S8. DOI: 10.1107/S1600577526009902/yi5197sup10.pdf

CCDC references: 2555130, 2555131, 2555132, 2555133, 2555134, 2555135, 2555136, 2555137

## Data Availability

Computed octahedral distortion parameters for all the datasets are available in the supporting information section and the CCDC deposition numbers for the datasets are 2555130 (FE4_80K_04_rotational_002), 2555131 (FE4_80K_04_meta_rotational_002), 2555132 (FE4_80K_04_meta_30min_rotational_001), 2555133 (FE4_80K_04_meta_60min_rotational_002), 2555134 (FE4_100K_01_rotational_001), 2555135 (FE4_100K_01_rotational_002), 2555136 (FE4_100K_01_Meta_rotational_001), and 2555137 (FE4_100K_01_Meta_rotational_002), which contain the supplementary crystallographic data for this paper, and can be obtained free of charge from the Cambridge Crystallographic Data Center via https://www.ccdc.cam.ac.uk/data_request/cif.
