# Supplementary material for: Steady-state diode-laser photocrystallography at the P11 beamline (PETRA III): proof-of-concept measurements on an Fe(II) metallogrid complex
Source: J Synchrotron Radiat. 2026 Oct 30;33(Pt 6):1718–26. doi: 10.1107/S1600577526009902 (PMC13753528; doi:10.1107/S1600577526009902)
Supplement: Supplementary file 1 [file s-33-01718-sup10.pdf]

JOURNAL OF SYNCHROTRON RADIATION

Volume 33 (2026)

Supporting information for article:

Steady-state diode-laser photocrystallography at the P11 beamline (PETRA III): proof-of-concept measurements on an Fe(II) metallogrid complex

Krishnayan Basuroy, Jose de Jesus Velazquez-Garcia, Sreeju Sreekantan Nair Lalithambika, Torben Reuss, Guillaume Pompidor, Alexander Grebentsov, Önder Akçaalan, Argha Barman and Simone Techert

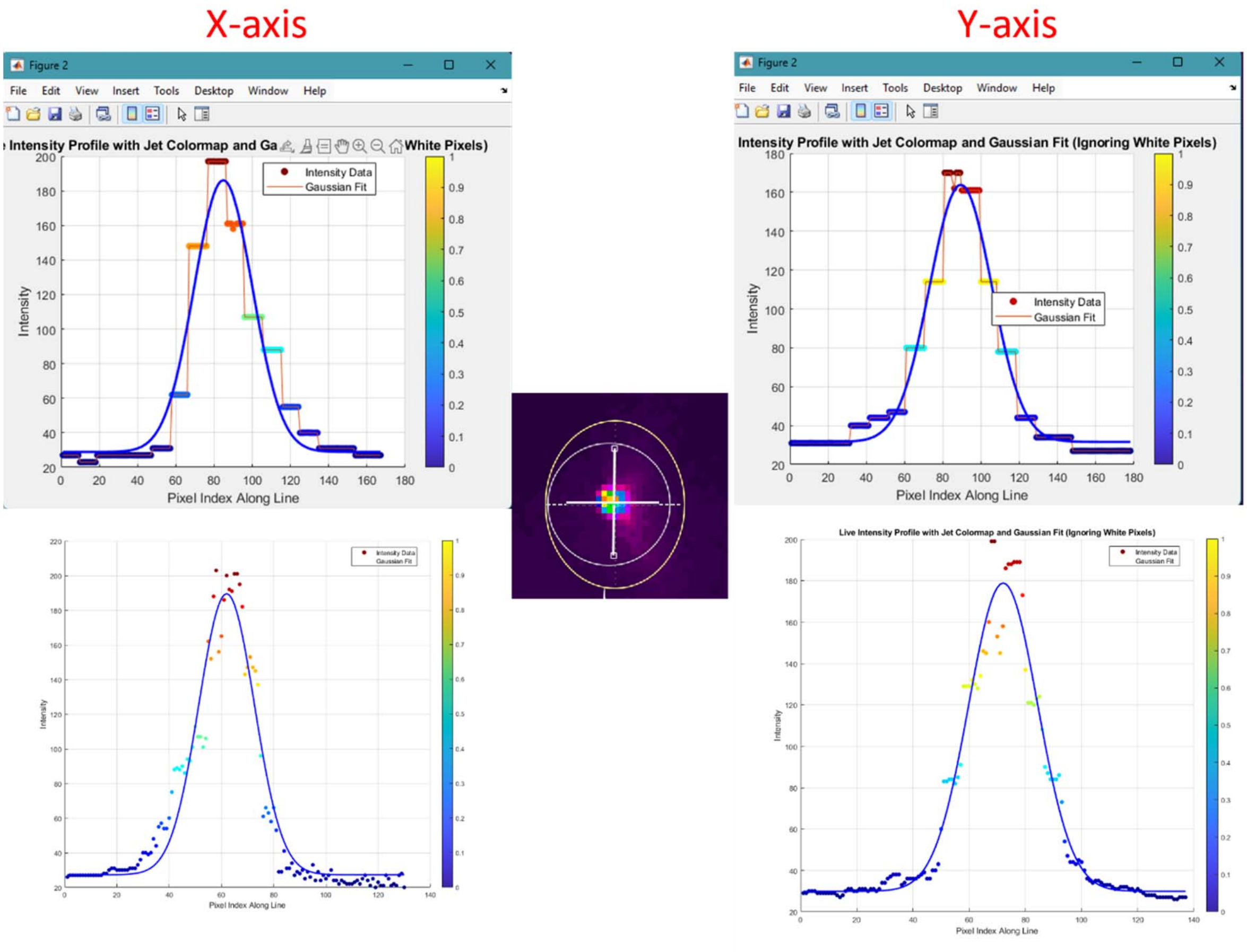

Figure S1 Beam cross-section of the laser pump beam was measured using an Ophir SP620U camera.

Table S1 Specification of the class 3B diode laser (taken from DeltaDiode Brochure provided by HORIBA Scientific).

Model	Peak Wave length	Spectral FWHM	Pulse Width Typical	Pulse Width Max	Peak Power	Average Power	Maximum Rep. Rate
DD-375L	375 ± 7 nm	<5nm	45 ps	70 ps	300 mW	1.8 mW	100 MHz

Table S2 Average power and average power density measured for a Delta Diode DD-375L diode laser.

Frequency of the laser (kHz)	***Without*** the focusing optics (μW)	***With*** the focusing optics (μW)	**Average power density** (μW/cm^2)	
			Without the focusing optics	***With*** the focusing optics
10	8.3	0.17	117.42	3554.20
20	8.4	0.21	118.84	4390.48
50	8.7	0.29	123.08	6063.05
100	9.2	0.45	130.15	9408.17
250	10.8	0.93	152.79	19443.56
500	13.4	1.71	189.57	35751.06
1000	18.6	3.27	263.14	68366.06
2000	29.1	6.43	411.68	134432.35
4000	50.3	12.03	711.60	251511.85
5000	60.9	15.98	861.56	334094.71
8000	92.9	25.7	1314.27	537311.27
10000	114.4	32.2	1618.43	673207.12
16000	182.0	52.5	2574.77	1097620.30
20000	221.0	64.4	3126.51	1346414.23
25000	290.0	85.1	4102.66	1779190.23
50000	555.0	166.0	7851.64	3470570.84
80000	870.0	263.0	12307.98	5498555.01

Table S3 Comparison of average Fe–N bond lengths and Fe···Fe distances before and after laser irradiation at 80 K and 100 K.

***Pre LASER Irradiation (PreLI)* (Å)**				***Post LASER Irradiation (PostLI)* (Å)**				***Δ (PostLI- PreLI)* (Å)**	
**$\langle Fe^{A}–N\rangle$**	**$\langle Fe^{B}–N\rangle$**	**$Fe^{A}\cdots Fe^{A'}$**	**$Fe^{B}\cdots Fe^{B'}$**	**$\langle Fe^{A}–N\rangle$**	**$\langle Fe^{B}–N\rangle$**	**$Fe^{A}\cdots Fe^{A'}$**	**$Fe^{B}\cdots Fe^{B'}$**	**$\Delta(Fe^{A}\cdots Fe^{A'})$**	**$\Delta(Fe^{B}\cdots Fe^{B'})$**
FE4_80K_04_rotational_002 (360° φ-scan)				***FE4_80K_04_meta_rotational_002(180° φ-scan)***					
**2.05±0.02**	**2.17±0.02**	**5.697±0.004**	**6.619±0.004**	**2.07±0.02**	**2.17±0.02**	**5.715±0.004**	**6.593±0.004**	**+0.018±0.006**	**–0.026±0.006**
				FE4_80K_04_meta_30min_rotational_001(360° φ-scan)					
				**2.06±0.02**	**2.17±0.02**	**5.731±0.005**	**6.575±0.004**	**0.034±0.006**	**-0.044±0.006**
				FE4_80K_04_meta_60min_rotational_002(360° φ-scan)					
				**2.05±0.02**	**2.17±0.02**	**5.691±0.004**	**6.628±0.003**	**-0.006±0.006**	**0.009±0.005**
FE4_100K_01_rotational_001(360° φ-scan)				***FE4_100K_01_Meta_rotational_001(360° φ-scan)***					
**2.05±0.02**	**2.19±0.02**	**5.762±0.004**	**6.617±0.004**	**2.05±0.02**	**2.19±0.02**	**5.787±0.004**	**6.631±0.003**	**0.025±0.006**	**0.014±0.005**
FE4_100K_01_rotational_002(180° φ-scan)				***FE4_100K_01_Meta_rotational_002(180° φ-scan)***					
**2.05±0.02**	**2.18±0.02**	**5.771±0.004**	**6.620±0.004**	**2.05±0.02**	**2.18±0.02**	**5.793±0.004**	**6.619±0.004**	**0.022±0.006**	**-0.001±0.006**

Table S4 Computed octahedral distortion parameters for all complexes.

FE4_80K_04_rotational_002

No. - Metal	<Fe-N>	ς	Δ	Σ	Θ
1 - Fe	2.0500	0.371810	0.001291	117.7372	377.7089
2 - Fe	2.1694	0.616124	0.002533	171.0815	703.2290
3 - Fe	2.0500	0.371811	0.001291	117.7372	377.7090
4 - Fe	2.1694	0.616124	0.002533	171.0815	703.2288

FE4_80K_04_meta_rotational_002

No. - Metal	<Fe-N>	ς	Δ	Σ	Θ
1 - Fe	2.0728	0.475134	0.001948	117.8752	362.8995
2 - Fe	2.1651	0.578355	0.002635	171.4526	703.3398
3 - Fe	2.0728	0.475133	0.001948	117.8751	362.8991
4 - Fe	2.1651	0.578356	0.002635	171.4526	703.3396

FE4_80K_04_meta_30min_rotational_001

No. - Metal	<Fe-N>	ς	Δ	Σ	Θ
1 - Fe	2.0592	0.329017	0.000949	115.2167	379.7903
2 - Fe	2.1690	0.280298	0.000594	174.4324	728.6171
3 - Fe	2.0592	0.329017	0.000949	115.2168	379.7904
4 - Fe	2.1690	0.280297	0.000594	174.4324	728.6170

FE4_80K_04_meta_60min_rotational_002

No. - Metal	<Fe-N>	ς	Δ	Σ	Θ
1 - Fe	2.0523	0.295949	0.000867	119.4740	375.4274
2 - Fe	2.1673	0.567338	0.002053	173.1538	719.7178
3 - Fe	2.0523	0.295949	0.000867	119.4739	375.4274
4 - Fe	2.1673	0.567336	0.002053	173.1537	719.7177

FE4_100K_01_rotational_001

No. - Metal	<Fe-N>	ς	Δ	Σ	Θ
1 - Fe	2.0472	0.404210	0.001387	113.8754	361.9729
2 - Fe	2.1887	0.473220	0.001611	169.8247	683.8576
3 - Fe	2.0472	0.404212	0.001387	113.8753	361.9732
4 - Fe	2.1887	0.473220	0.001611	169.8247	683.8577

FE4_100K_01_meta_rotational_001

No. - Metal	<Fe-N>	ς	Δ	Σ	Θ
1 - Fe	2.0521	0.238745	0.000676	104.1359	347.6072
2 - Fe	2.1891	0.374751	0.001038	170.0408	693.5204
3 - Fe	2.0521	0.238747	0.000676	104.1358	347.6072

4 - Fe	2.1891	0.374752	0.001038	170.0409	693.5206

FE4_100K_01_rotational_002

No. - Metal	<Fe-N>	ς	Δ	Σ	Θ
1 - Fe	2.0448	0.299568	0.000885	105.9672	340.9529
2 - Fe	2.1799	0.462694	0.001643	172.5449	698.1172
3 - Fe	2.0448	0.299567	0.000885	105.9672	340.9529
4 - Fe	2.1799	0.462694	0.001643	172.5449	698.1173

FE4_100K_01_meta_rotational_002

No. - Metal	<Fe-N>	ς	Δ	Σ	Θ
1 - Fe	2.0455	0.230481	0.000602	110.6002	346.1760
2 - Fe	2.1784	0.421520	0.001299	173.7888	715.1964
3 - Fe	2.0455	0.230481	0.000602	110.6002	346.1761
4 - Fe	2.1784	0.421520	0.001299	173.7888	715.1964

* The term “Meta” identifies the datasets collected post laser irradiation.

Table S5 The estimated relative photoconversion indicator, calculated from the Fe-N bond length changes at the site A (LS site).

**Data sets**	**%ΔX_{HS}**
FE4_80K_04_Meta_rotational_002	~23
FE4_80K_04_Meta_30min_rotational_001	~9

Table S6 Propagated uncertainty analysis for changes in Fe···Fe separations before and after laser irradiation.

**Comparison**	**Parameter**	**Pre laser irradiation / Å**	**Post laser irradiation / Å**	**Δ / Å**	**σΔ / Å**	**Δ/σΔ**	**Interpretation**
80 K post 2–3 min − pre	FeA···FeA′	5.697 ± 0.004	5.715 ± 0.004	+0.018	0.006	+3.2	significant expansion
80 K post 2–3 min − pre	FeB···FeB′	6.619 ± 0.004	6.593 ± 0.004	−0.026	0.006	−4.6	significant contraction
80 K post 30 min − pre	FeA···FeA′	5.697 ± 0.004	5.731 ± 0.005	+0.034	0.006	+5.3	significant expansion
80 K post 30 min − pre	FeB···FeB′	6.619 ± 0.004	6.575 ± 0.004	−0.044	0.006	−7.8	significant contraction
80 K post 60 min − pre	FeA···FeA′	5.697 ± 0.004	5.691 ± 0.004	−0.006	0.006	−1.1	not significant / relaxed
80 K post 60 min − pre	FeB···FeB′	6.619 ± 0.004	6.628 ± 0.003	+0.009	0.005	+1.8	not significant / weak
100 K set 1 post 2–3 min − pre	FeA···FeA′	5.762 ± 0.004	5.787 ± 0.004	+0.025	0.006	+4.4	significant expansion
100 K set 1 post 2–3 min − pre	FeB···FeB′	6.617 ± 0.004	6.631 ± 0.003	+0.014	0.005	+2.8	significant expansion, but not contraction and opposite in direction to the 80K contraction
100 K set 2 post 2–3 min − pre	FeA···FeA′	5.771 ± 0.004	5.793 ± 0.004	+0.022	0.006	+3.9	significant expansion
100 K set 2 post 2–3 min − pre	FeB···FeB′	6.620 ± 0.004	6.619 ± 0.004	−0.001	0.006	−0.2	not significant

Table S7 Average equivalent isotropic B factors calculated from U_{eq} values for selected atom groups in the pre laser irradiation and post laser irradiation datasets.

**Mean B factors (Å^2) from CIF files**						
**Dataset**	**Fe atoms**	**N atoms**	**Fe+N core**	**Framework non-H excluding BF_4**	**BF_4 only**	**All non-H**
FE4_80K_04_rotational_002 (**A**)	5.57	9.02	8.53	9.55	18.93	10.86
FE4_80K_04_meta_rotational_002 (**B**)	8.09	11.84	11.31	12.69	23.36	14.17
FE4_80K_04_meta_30min_rotational_001 (**C**)	6.28	9.70	9.21	10.15	21.18	11.68
FE4_80K_04_meta_60min_rotational_002 (**D**)	6.47	10.08	9.57	10.77	20.64	12.14
FE4_100K_01_rotational_001 (**E**)	3.43	5.97	5.61	6.49	15.29	7.72
FE4_100K_01_meta_rotational_001 (**F**)	5.25	7.74	7.38	8.47	18.97	9.93
FE4_100K_01_rotational_002 (**G**)	4.58	7.41	7.00	8.11	17.24	9.38
FE4_100K_01_meta_rotational_002 (**H**)	6.24	9.32	8.88	9.91	19.72	11.27
**Change relative to corresponding pre laser irradiation dataset**						
**Comparison**	**ΔFe atoms**	**ΔN atoms**	**ΔFe+N core**	**Δframework non-H excluding BF_4**	**ΔBF_4 only**	**Δall non-H**
**B – A** (Å^2)	+2.53	+2.82	+2.78	+3.14	+4.42	+3.32
**C – A** (Å^2)	+0.71	+0.68	+0.68	+0.60	+2.24	+0.83
**D – A** (Å^2)	+0.91	+1.06	+1.04	+1.22	+1.71	+1.29
**F – E** (Å^2)	+1.82	+1.77	+1.78	+1.98	+3.69	+2.21
**H – G** (Å^2)	+1.66	+1.91	+1.88	+1.79	+2.48	+1.89

Table S8 Unit-cell and selected data-quality/refinement indicators.

Code	**Condition**	T (K)	Delay	Scan	Cell volume (Å³)	ΔV vs pre (%)	Resolution, dmin (Å)	Completeness (%)	Rint	R1	wR2
A	Pre laser irradiation	80	—	360° φ	9042.4(5)	—	1.261	70.5	0.0392	0.1689	0.5189
B	Post laser irradiation	80	2–3 min	180° φ	9065.7(5)	+0.258	1.341	77.9	0.0306	0.1588	0.4505
C	Post laser irradiation	80	30 min	360° φ	9040.8(5)	−0.018	1.518	92.9	0.0410	0.1607	0.4593
D	Post laser irradiation	80	60 min	360° φ	9075.6(5)	+0.367	1.273	71.0	0.0560	0.1545	0.4502
E	Pre laser irradiation, set 1	100	—	360° φ	9065.0(5)	—	1.272	65.3	0.0439	0.1491	0.4304
F	Post laser irradiation, set 1	100	2–3 min	360° φ	9096.3(5)	+0.345	1.320	69.1	0.0351	0.1480	0.4184
G	Pre laser irradiation, set 2	100	—	180° φ	9074.2(5)	—	1.320	69.6	0.0330	0.1514	0.4194
H	Post irradiation, set 2	100	2–3 min	180° φ	9102.9(5)	+0.316	1.274	65.0	0.0399	0.1524	0.4478

Codes A–H correspond to the same dataset definitions used in Table S7.

The resolution was calculated from the CIF values using $d_{min}=\lambda/[2\sin(\theta_{max})]$, with $\lambda=0.82655$Å.

The cell volume does **not** follow the time-dependent Fe···Fe response at 80 K:

- At 2–3 min, the volume increases by 0.258%.
- At 30 min, when the coupled FeA···FeA′ expansion and FeB···FeB′ contraction is strongest, the volume is nearly unchanged in relative terms: −0.018%.
- At 60 min, after the Fe···Fe response has substantially relaxed, the volume shows its largest increase: +0.367%.

Moreover, the two 100 K post laser irradiation datasets show volume increases of +0.345% and +0.316%, even though the characteristic coupled 80 K Fe···Fe response is not reproduced. Therefore, a small increase in unit-cell volume cannot by itself explain the specific 80 K grid distortion.

The diffraction/refinement indicators also do not show progressive deterioration with post laser irradiation delay. At 80 K, R1 and wR2 are actually lower in all three post laser irradiation datasets than in the pre laser irradiation dataset. Completeness and resolution differ between datasets because the angular coverage, scan extent and resolution limits were not identical.

The absolute R1 and wR2 values remain high and the completeness is limited, so this analysis does **not prove** that radiation damage or heating is absent. It does show that there is no simple correlation between progressive diffraction deterioration, uniform cell expansion and the characteristic coupled Fe···Fe response at 80 K.
